# Why Is Maternal Control Harmful? The Relation between Maternal Control, Insecure Attachment and Antisocial Personality Disorder Features in Chinese College Students: A Sequential Mediation Model

**DOI:** 10.3390/ijerph191710900

**Published:** 2022-09-01

**Authors:** Hui Ling, Fanfei Meng, Yaqin Yan, Hong Feng, Jianren Zhang, Linrui Zhang, Siyang Yuan

**Affiliations:** 1 Department of Psychology, Hunan Normal University, Changsha 410081, China; 2 School of Preschool Education, Changsha Normal University, Changsha 410100, China; 3 Department of Student Affairs, Hunan First Normal University, Changsha 410205, China; 4 Hunan Wenjin Research Institute of Education, Changsha 410013, China; 5 Cognition & Human Behavior Key Laboratory of Hunan Province, Hunan Normal University, Changsha 410081, China; 6 Department of Psychology, University of Toronto Scarborough, Toronto, ON M4Y 1M7, Canada; 7 School of Dentistry, University of Dundee, Dundee DD1 4HN, UK

**Keywords:** antisocial personality disorder, maternal control, parental antipathy and neglect, adverse childhood experience

## Abstract

Background: Previous work has indicated that a negative parenting style is associated with antisocial personality disorder features in Chinese college students, yet few studies have explored the unique role of negative mothering in children’s antisocial personality disorder. Methods: The current study mainly examined the sequential mediation effect of parental antipathy and neglect (PAN) and mother negative loving (a form of insecure attachment) in the association between mother control and adulthood antisocial personality disorder features (ASPD features) in the framework of attachment theory and cognitive–behavioral theory. A community sample of 1547 Chinese college students filled in the Parental Bonding Instrument, the Childhood Experience of Care and Abuse Questionnaire, the Adult Attachment Questionnaire and the Personality Diagnostic Questionnaire-4+. Results: A sequential mediation model analysis showed that maternal control significantly predicted PAN, mother negative loving, as well as ASPD features. Conclusions: Mother control and mother negative loving appear to advance on the development and exacerbation of ASPD features in college students.

## 1. Introduction

Antisocial personality disorder (ASPD) is a pattern of personality disorder which is characterized by dysfunctional interpersonal relationships and shows impulsivity, aggression and lack of empathy or remorse [1,2]. According to the *Diagnostic and Statistical Manual of Mental Disorders-Fifth Edition* (DSM-5) [3], ASPD begins in childhood or early adolescence and continues into adulthood [4,5]. It is reported that the prevalence of ASPD is 2–3% in the general population [6]. According to past research, personality disorders (PDs) are widespread in college students [7]. Among the PDs, ASPD has strong criminal tendencies, therefore, ASPD is a potential factor endangering campus and social security in China [8].

The maltreatment-ASPD association has been a focus of research for decades [4]. Multiple high-risk factors are related to maltreatment, such as strict and ineffective parenting styles [9], insecure attachment [10,11], negative family ecology [12] and early negative experiences [13,14,15], which are all adverse childhood experiences (ACEs). The ACEs include a variety of types of trauma, including psychological/physical abuse; violence in the home; living with a mentally ill or suicidal person; living in poverty; and living with a substance abuser [16,17]. A recent study has found that ACEs were related to PDs [18], but how do ACEs contribute to ASPD in adulthood?

According to the cognitive-behavior theory of PDs [19], ASPD contains a distinct maladaptive cognitive profile [20,21]. ASPD individuals view themselves as loners and autonomous; some see themselves as having been mistreated by others and therefore justify victimizing others for believing that they have been victimized. Meanwhile, they believe that their offensive peers deserve being humiliated [20].

Young et al. (2003) [21] have stated that early maladaptive cognitive schema of ASPD develops in childhood in response to genetic predisposition and environmental influences, where parenting style plays a prominent role. According to the coercive family process theory [22], negative parent–child interaction contributes to children’s aggressive behaviors [23]. Furthermore, studies indicate that early negative parenting experience correlates to adulthood ASPD [2,24].

It has been noted that the mother figure is different from the father figure in parenting in Chinese culture [25,26,27]. Compared with fathers, mothers provide less discipline but more warmth and love which is more critical to a safe parent-child bond [27,28,29,30]. Therefore, as attachment theory suggests [31], children are more likely to establish attachment relationships with their mothers. In addition, mothers’ negative parenting can easily damage the mother–child bond [32]. Nevertheless, few studies have explored the unique role of the mother figure in the association between adverse childhood experience and ASPD. Although the influence maternal mental health has on children’s ASPD has been explored [24], few studies have studied the unique associations between negative maternal parenting style and children’s ASPD. Meanwhile, from the perspective of the integration of attachment theory and family process theory, no research has yet examined the effects of maternal control, perceived parental neglect and abuse, and parent-child attachment on ASPD in children.

## 2. The Establishment of Theoretical Hypothesis Models

### 2.1. PAN in the Association between Maternal Control and ASPD Features

According to coercive family theory [22], interpersonal processes within the family are a causal mechanism in the emergence and escalation of violent behavior. Early harsh parenting in response to child misbehavior serves as a role model by which children initially learn to deal with interpersonal disagreements in a confrontational, aggressive manner [23]. As an invalidating maternal parenting style, maternal control refers to the mother’s intrusion and encouragement of psychological dependence towards the child [33,34,35], and may cause children to develop maladaptive cognitive schema. Self-cognition schema significantly mediates the relationship between negative parenting style and negative psychological consequences for children [36]. Similarly, children who form a mother-schema that mothers are uncaring and unloving and a self-schema that “I have been abused” may treat others and society in the same intrusive and vindictive way as they learned from their perceived maternal control [30], which is consistent with the cognitive-behavior theory of ASPD [19].

The ACE framework has consistently shown that more significant and more diverse exposure to various forms of abuse, neglect and childhood adversity is associated with expansive mental health and behavioral problems across the lifespan [16,17,37]. Research evidence suggests that childhood abuse indicates an individual’s adulthood ASPD [4,5,38]. As a typical type of abuse, mental abuse refers to the neglect and antipathy of the primary caregiver perceived by children [39] and was found to be positively related to ASPD [13,40,41]. The abuse-ASPD link can be explained by cognitive–behavioral theory; that is, individuals who perceived childhood abuse would believe that they have been victimized and justify victimizing others, and then they consider others as exploitative and thus deserving of being abused in retaliation [20].

How does maternal control harm children? Given the discussion mentioned above, we intend to establish a mediation model in which PAN mediates the association between maternal control and adulthood ASPD. The internal working model of attachment theory [42,43] specified that repeated experiences of interacting with the mother generate a system of thoughts, memories, beliefs, expectations, behaviors and related emotions about the self, the others and self–other relationships, and this internalized working model could bear a profound impact on an individual in social contexts across his or her lifetime [44].

### 2.2. Mother Negative Loving in the Association between Maternal Control and ASPD

Mother negative loving refers to a mother–child insecure attachment caused by children’s failure to feel maternal love [45]. Bowlby (1988) [46] proposes that attachment insecurity is produced when an individual experiences his or her attachment needs as not being routinely met, which promotes the development of internal working models of the self as unlovable and others viewed as undependable and unsupportive [10]. Maternal control ignores or even deprives children’s needs for personal will and autonomy, which may pose a threat to the mother–child attachment as well as the healthy development of children’s personality [47]. Hence, it is reasonable to assume that maternal control is positively correlated with mother negative loving.

The feeling of lack of maternal love accelerates the formation of a negative internal working model [45,46] with a maladaptive other-schema: others do not love me and are unfriendly to me [48], which is consistent with the cognitive characteristics of ASPD [20]. Furthermore, Ainsworth and Bowlby (1991) [49] considered that the central tenet of attachment theory is that early interactions with caregivers shape the development of personality throughout life, and several studies provided support for this argument [11,50]. Therefore, based on the above evidence, it is reasonable to believe that maternal control parenting may predict students’ ASPD through the insecure mother–child attachment.

### 2.3. Linking PAN to Mother Negative Loving

Childhood abuse experience is considered as one of the most prominent causes of insecure adult attachment [51]. Children who have been abused (antipathy and neglect) were more likely to develop mother–child insecure attachment [52]. From the perspective of the attachment developing, parental abuse not only fails to satisfy children’s attachment needs, but also inflicts harm and fear on children, which is a great deprivation of children’s sense of security [53,54]. As a result, we believe that participants who perceive PAN may feel mother negative loving.

### 2.4. Sequential Mediation Effect of PAN and Mother Negative Loving

With the benefit of previous research, we found clues to establish associations between these study variables. For example, an insecure attachment was found to mediate the association between childhood abuse and borderline personality disorder [55]. Cohen et al. (2016) [53] further found that childhood abuse caused individuals’ attachment anxiety, which in turn leads to their poor self-control and difficulty in getting along in harmony with others. This is consistent with specific characteristics of ASPD [56]. Therefore, based on the two mediation models with PAN and mother negative loving mediating the association between maternal control and ASPD, we further established an integration model to explore the complex association between the four variables. Of note, to be in line with cognitive behavior theory [19], the three predictors were all considered cognitive variables which were assessed by recalling and perceiving the childhood experience.

### 2.5. The Current Study

**Figure 1:**
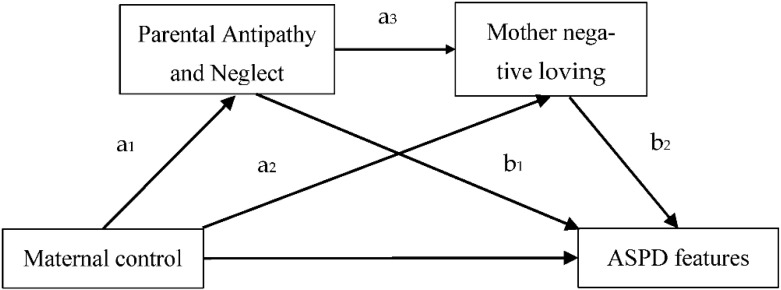
A sequential mediation model of maternal control, PAN, mother negative loving and ASPD. Note. The a_1_, a_2_, a_3_, b_1_ and b_2_, respectively, refer to the regression coefficients of different paths in this sequential mediation model.

In the current study, we propose a conceptual model that attempts to integrate early experiences of maternal negative parenting with subsequent cognitive–affective processes involved in the development and maintenance of ASPD features. Specifically, it suggests that early experiences of maternal control contribute to the development of ASPD features via parental antipathy and neglect (PAN) and mother negative loving. The primary aim of our study is to delineate this conceptual model in more detail and provide preliminary data to support it in a non-clinical Chinese college students sample. To achieve this goal, in the framework of the cognitive–behavioral theory of ASPD, we built a sequential mediation model which integrated the perceived maternal control, PAN and mother negative loving (see Figure 1). Specifically, in order to examine the underlying mechanisms of ASPD, we put forward the following hypotheses:

**Hypotheses 1** **(H1).** *PAN would mediate the association between maternal control and ASPD*.

**Hypotheses 2** **(H2).** *Mother negative loving would mediate the association between maternal control and ASPD*.

**Hypotheses 3** **(H3).** *PAN and mother negative loving would sequentially mediate the relationship between maternal control and college students’ ASPD*.

## 3. Materials and Methods

### 3.1. Participants

Participants were recruited from seven universities in Changsha, Hunan Province, China. After data cleansing was administered, a total of 1547 valid cases were obtained. The mean age of the participants was 19.77 years (range = 17–30 years, SD = 1.23). Among this sample, 846 were male, 697 were female and 4 had “gender” missing. Furthermore, 480 were the only child in the family, 972 had siblings in the family and 95 did not indicate information about siblings.

### 3.2. Measures

Antisocial personality disorder features (ASPD features) were measured using the 107-item Personality Diagnostic Questionnaire-4+ (PDQ-4+), which is designed to assess 12 patterns of personality disorders in the DSM-IV. Participants report on this dichotomous measurement with “true = 1” or “false = 0”. Yang et al. (2002) [57] revised it in the Chinese context. The revised version has better reliability and validity, high sensitivity and moderate specificity for personality disorder screening. In this study, a score of 5 was used as the criterion for screening college students with ASPD; Cronbach’s alpha for the scale was 0.75; and the prevalence of ASPD was 3.7% (n = 58) in our sample of Chinese college students.

Parental antipathy and neglect were measured using antipathy and neglect sub-scale from the Childhood Experience of Care and Abuse Questionnaire (CECA.Q) [39,58]. The scale comprises sixteen items: eight relating to antipathy (e.g., “He/she was very difficult to please”) and eight relating to neglect (e.g., “He/she was concerned about my worries”). Through retrospective recall, participants felt and assessed the antipathy or neglect from their parents on every item. Items were scored as 1 for “yes definitely” to 5 for “not at all”. The two scales are repeated for mother and father figures. High scores indicate that individuals perceive more PAN. This Chinese version was considered to have good reliability and validity [59].

Maternal control, an invalidating parenting style, was measured using the Parental Bonding Instrument—Chinese version [60]. The PBI mainly assesses the attitudes and behaviors of parents perceived by children during the first 16 years of life [61]. The PBI-C consists of three factors (i.e., care, encourage autonomy and control) [60]. High scores in control indicate over-protection, intrusion and encouragement of psychological dependence, and low scores suggest the permission of independence and autonomy [61]. Research showed that this revised version has good reliability and validity [60].

Mother negative loving was measured by six items that reflect an insecure mother–child attachment from the Adult Attachment Questionnaire (AAQ 3.1) [42]. Participants self-reported on the items (e.g., In my childhood, my mother made me feel that she did not like me around; In my childhood, my mother was too busy to accompany me) to assess, as much as possible, the mother–child affective bond perceived by individuals by recalling and evaluating their situation during the period around the age of six. Items were scored as 1 for “strongly disagree” to 5 for “strongly agree”, and a high score indicates that the participant perceives an insecure attachment relationship with his/her mother. The AAQ 3.1 has good reliability and validity [42]. In this study, Cronbach’s alpha for this scale was 0.67.

### 3.3. Procedure

The study was approved by the Ethical Committee for Scientific Research in the Hunan Normal University and has been executed in conformity with ethical standards laid down in the 1964 Declaration of Helsinki and its later amendments [62]. College students were recruited by researchers and research assistants and welcomed to complete a questionnaire survey. All participants were informed of the voluntary nature of the investigation. Prior to study participation, the researcher clarified any ethical issues and all participants gave written informed consent.

### 3.4. Data Analysis

Firstly, we deleted the extreme values beyond the range of ±3 standard deviations and replaced missing values with the average value. Secondly, Hayes’s (2013) [63] PROCESS macro (model 6) in SPSS was used to investigate the sequential mediation effect of PAN and mother negative loving between maternal control and ASPD. Bias-corrected bootstrapping, based on 5000 samples, was used to estimate the indirect effect’s standard error. A 95% confidence interval (CI) was used to examine the significance of the mediation effect. To minimize multi-collinearity, all the predictors were standardized. Given that age, gender [64], only child status and place of residence [65] might be correlated with ASPD [66], we controlled these demographic variables in our statistical analyses.

## 4. Results

### 4.1. Descriptive Statistics

Table 1 presents the means, standard deviations and correlations for the measured variables. As expected, maternal control, PAN, mother negative loving and ASPD were positively related to each other (all *p* < 0.01).

### 4.2. Testing for Sequential Mediation Effect

Next, we tested the sequential mediation effect of PAN and mother negative loving in the association between maternal control and ASPD features. As seen in Table 2, results indicate that the total effect of maternal control on ASPD was significant (*β* = 0.13, *SE* = 0.03, *t* = 5.22, *p* < 0.001). However, the direct effect without the mediating effects of PAN and mother negative loving was also found to be significant (*β* = 0.09, *SE* = 0.03, *t* = 3.29, *p* = 0.001).

Results of regression equations testing mediation models are presented in Table 3 and shows that maternal control (*β* = 0.09, *SE* = 0.03, *p* < 0.01), PAN (*β* = 0.06, *SE* = 0.03, *p* < 0.05) and mother negative loving (*β* = 0.09, *SE* = 0.03, *p* < 0.01) significantly predict ASPD in adulthood, and the three predictors explain 4% of the variance in ASPD features. Maternal control (*β* = 0.20, *SE* = 0.02, *p* < 0.001) and PAN (*β* = 0.44, *SE* = 0.02, *p* < 0.001) were positively associated with mother negative loving, and the R^2^ value shows that the model explains 27% of the variance in mother negative loving. Maternal control was positively associated with PAN (*β* = 0.26, *SE* = 0.03, *p* < 0.001), and the R^2^ value depicts that maternal control explains 8% of the variance in PAN.

Moreover, specific indirect effects through PAN (a_1_b_1_ = 0.02, *SE* = 0.01, 95% CI = [0.001+, 0.03]) and mother negative loving (a_2_b_2_ = 0.02, *SE* = 0.01, 95% CI = [0.01, 0.03]) were both found to be significant. Hence, Hypotheses 1 and 2 were supported. Finally, while testing for sequential mediation, a specific indirect effect of maternal control on ASPD of Chinese college students, with both mother negative loving and PAN in the model, was also found to be significant (a_1_a_3_b_2_ = 0.01, 95% CI = [0.003 ^+^, 0.02]., hence providing support for Hypotheses 3. It means that our theoretical model was supported by the data (See Table 4).

## 5. Discussion

### 5.1. Main Findings

The current study proposed a sequential mediation model to investigate how maternal control in childhood influenced adulthood ASPD features. The results showed that PAN and mother negative loving sequentially mediated the association between maternal control and ASPD features.

First, the prevalence of ASPD was 3.7% in the Chinese college student sample, which is consistent with the prevalence of 3.6% in an epidemiological study report [6]. This was, however, much higher than the prevalence in a study by Goldstein [67]. Goldstein stated that the screening measurement with a looser standard and the participants’ cover-up answers might lead to lower prevalence results. Therefore, it is reasonable to believe that our result is credible and should be taken seriously for college campus safety and social stability [8].

In general, we found a tight ACEs–ASPD association in our study, which is consistent with previous studies [17,18]. Werner et al. found that ASPD has a common genetic basis, which makes ASPD in different backgrounds show similar patterns [68]. The correlation analysis results depicted that ASPD features were positively correlated to maternal control, PAN and mother negative loving. Individuals who perceived more maternal control, PAN and mother negative loving would be more likely to be suffered from ASPD features. However, Batool et al. (2017) [9] found that parental control and ASPD were significantly uncorrelated. The main reason for this discrepancy may be due to the fact that the overall effects of maternal control and paternal control were calculated simultaneously [9]; maternal control was found to be different to paternal control with adolescents [69]. Given that few studies have explored the relationship between paternal control and ASPD features in early adulthood, we plan to explore this issue further.

Second, results showed that PAN partially mediated the association between maternal control and ASPD features, indicating that maternal control, as an invalidating parenting style, may bring feelings of neglect and antipathy. Also, the perceived PAN may contribute to ASPD features in adulthood. Just as the coercive family model indicates, parents’ negative reaction to children will increase their behavioral problems [70]. Our findings supported this theory model and initially revealed the process mechanism by which parents’ negative feedback leads to adulthood ASPD features.

Third, we also found that mother negative loving mediates the association between maternal control and ASPD features. This manifested that maternal control may lead to mother–child insecure attachment which mainly derives from the lack of maternal love perceived by children. In turn, the mother–child insecure attachment expressed by mother negative loving may cause ASPD features in adulthood. Therefore, this result provides more detailed and powerful support for attachment theory [71,72]. The abuse experiences in early childhood attachment will make the individual form a negative impression of others, and they would perceive the outside world to be unsafe, and others do not love them. With this internal working model, children will develop more aggressive or spiteful attitudes and behaviors [11].

Results also indicated that PAN might lead to mother negative loving perceived by children, indicating that maltreatment from both parents experienced by children may damage the child’s attachment to the mother. This result was consistent with a meta-analysis study which found a robust relationship between ACE and insecure attachment [18,51]. Attachment theory can also explain that primary caregivers’ response to a child’s attachment needs is the key to children’s development of a sense of security, which is a sign of a secure attachment for children [32].

More importantly, results supported the hypothesis that PAN and mother negative loving sequentially mediated the association between the maternal control and ASPD features. Specifically, maternal control parenting style brought the perception of PAN to children which, in turn, lead to insecure attachment, and ultimately laid hidden dangers for ASPD in adulthood. Of note, the main predictors were assessed by college students’ retrospective negative experiences in childhood; that is, participants’ response to maternal parenting style, PAN and mother negative loving were mainly carried out through perceiving, which mainly reflects the cognitive component of these variables. From this perspective, the cognitive–behavioral theory of ASPD [19] can provide more practical information: individuals with ASPD may perceive more maltreatment from others and society, and they are more inclined to think that others do not care about them or will even harm them [20].

### 5.2. Limitations and Advantages

Several limitations of this study should be acknowledged. First, the current study used a retrospective data collection method in a cross-sectional design. Although, logically, we suggest that the recalled childhood maternal control and insecure mother–child attachment influences later ASPD features in adulthood, the possibility of their mutual influence cannot be completely ruled out. Specifically, adults with ASPD features may be more prone to recall more memories and emotions of early poor maternal parenting. Meanwhile, the self-report response can hardly rule out subjective and social praise effects, which may interfere with the authenticity of the participants’ responses. Therefore, future research should incorporate a longitudinal study design and more objective measurements (parental self-evaluation) to assess the real, rather than recalled, perceived maternal control and mother child attachment. Next, the predictor variables measured in the study contain not only cognitive components, but also involve complex emotional responses [53], which we have not yet considered. Therefore, future researchers should adopt more appropriate cognitive variables, such as self-schema or other-schema [72], to test the ASPD the cognitive–behavioral model and reveal the cognitive process mechanism of the association between maternal control and ASPD features. Also, we studied ASPD with a non-clinical sample of college students, so we are actually analyzing the ASPD characteristics of college students. Therefore, in the future, it would be better for us to study ASPD clinical samples to better reveal the pathogenic mechanism of ASPD. Last but not the least, in current study, we mainly focused on the maternal parenting without discussing the paternal role. Therefore, the fathers’ role in the family, as well as the interaction between the fathers and mothers should be measured and discussed in the future. Meanwhile, we should broaden our horizons to study those protective factors in children’s future lives, for example, the social support [73]. Also, we need to include more negative factors within the framework of ACEs to uncover the pathogenic mechanism of ASPD.

Despite the limitations, the current study had theoretical and practical significance. First, the results support the coercive family theory and attachment theory and provide evidence to explain how PAN and mother negative loving mediates the relationship between maternal control and college students’ ASPD. Results of the study not only reveal the cognitive process through which mother control leads to ASPD but highlight the effect the mother figure has on children’s personality consequences. These findings have at least two implications for the prevention and treatment of college students’ ASPD features. On the one hand, these findings highlight the harmful effect of maternal control because it may lead to children’s PAN feelings, lacking mother love and adulthood ASPD features. It may be helpful to support parenting styles focused on reducing maternal control and increasing maternal autonomy support. Furthermore, this result also suggests that mothers should unconditionally support their children and create a warm and loving psychological atmosphere for their children. On the other hand, given that negative cognitive processes (perception of PAN and mother negative loving) mediate maternal control and ASPD features, clinical interventions should better focus on replacing the cognitive schema or pattern of children who experienced maternal control, such as the unlovable or abused self and indifferent others, with a positive cognitive schema of lovable self and caring others [20].

## 6. Conclusions

In the framework of cognitive–behavioral theory, we integrated the coercive family model and attachment theory. We mainly focused on the unique influence of the mothers’ role has on students’ ASPD features. From this study, the perceived negative experience may exert a continuous influence on children’s personalities. Consequently, the current study explored the impact of early adverse experience on children’s mental health and elucidated a potential cognitive process mechanism connecting recalled early experiences (namely, maternal caregiving) and current psychopathology (namely, antisocial personality features).

## Figures and Tables

**Table 1:** Means, standard deviations and correlations of the study variables.

	1	2	3	4	5	6	7	8
1. Age	—							
2. Gender	0.07 **	—						
3. Place of residence	−0.01	−0.02	—					
4. Only-child	−0.11 **	0.04	−0.16 **	—				
5. MC	−0.06 *	0.07 **	−0.01	0.14 **	—			
6. MNL	0.00	−0.00	0.00	−0.04	0.30 **	—		
7. PAN	0.05	0.03	−0.01	−0.09 **	0.24 **	0.49 **	—	
8. ASPD features	−0.02	0.07 **	−0.03	0.02	0.14 **	0.15 **	0.13 **	—
*M*	19.78	0.55	0.54	0.37	5.14	11.45	68.06	0.04
*SD*	1.13	0.50	0.48	0.44	3.31	3.88	16.49	0.19

Note. N = 1547. MC = maternal control. MNL = mother negative loving. PAN = parental antipathy and neglect. DV= dependable variable. * *p* < 0.05. ** *p* < 0.01.

**Table 2:** Total effect and direct effect.

	Total Effects (DV = ASPD Features)			Direct Effect (DV = ASPD Features)		
	*β* (*SE*)	*t*	*p*	*β* (*SE*)	*t*	*p*
MC	0.13 (0.03)	5.22 ***	0.00	0.09 (0.03)	3.29 **	0.001

Note. N = 1547. MC = maternal control. ASPD = antisocial personality disorder. DV = dependable variable. *SE* = standard error. ** *p* < 0.01, *** *p* < 0.001.

**Table 3:** All regression equations testing mediation models with maternal control as the independent variable.

	Model 1 (DV: PAN)			Model 2 (DV: MNL)			Model 3 (DV: ASPD Features)		
	*β* (*SE*)	*t*	*p*	*β* (*SE*)	*t*	*p*	*β* (*SE*)	*t*	*p*
MC	0.26 (0.03)	10.45 ***	0.00	0.20 (0.02)	8.57 ***	0.00	0.09 (0.03)	3.29 **	0.001 ^+^
PAN				0.44 (0.02)	19.33 ***	0.00	0.06 (0.03)	2.11 *	0.04
MNL							0.09 (0.03)	3.21 **	0.001 ^+^
*R* ^2^	0.08			0.27			0.04		
*F*	25.68 ***			95.45 ***			9.13 ***		

Note. N = 1547. MC = maternal control. MNL = mother negative loving. PAN = parental antipathy and neglect. DV= dependable variable. * *p* < 0.05. ** *p* < 0.01. *** *p* < 0.001. ^+^ three decimal places in the value are reserved.

**Table 4:** Bootstrapping point estimates and 95%CIs for all indirect effects with maternal control as the independent variable.

	Effect	*SE*	Bootstrapping 95% CI	
			Lower	Upper
a_1_b_1_	0.02	0.01	0.001 ^+^	0.03
a_2_b_2_	0.02	0.01	0.01	0.03
a_1_a_3_b_2_	0.01	0.004 ^+^	0.003 ^+^	0.02
Total indirect effect	0.05	0.01	0.02	0.07

Note. N = 1547. *SE* = standard error. CI = Confidence interval. a_1_b_1_ = MC → PAN → ASPD features. a_2_b_2_ = MC → MNL → ASPD features. a_1_a_3_b_2_ = MC → PAN → MNL → ASPD features. MC = maternal control. MNL = mother negative loving. PAN = parental antipathy and neglect. DV= dependable variable. ^+^ three decimal places in the value are reserved.

## Data Availability

The data presented in this study are available upon request from the corresponding author. The data are not publicly available.
